# Methodological quality of 100 recent systematic reviews of health-related outcome measurement instruments: an overview of reviews

**DOI:** 10.1007/s11136-024-03706-z

**Published:** 2024-07-03

**Authors:** Ellen B. M. Elsman, Lidwine B. Mokkink, Inger L. Abma, Olalekan Lee Aiyegbusi, Alessandro Chiarotto, Kirstie L. Haywood, Karen Matvienko-Sikar, Daniella M. Oosterveer, Jan J. M. Pool, Ilse E. J. Swinkels-Meewisse, Martin Offringa, Caroline B. Terwee

**Affiliations:** 1 Department of Epidemiology & Data Science, Amsterdam UMC, Amsterdam Public Health Research Institute, Vrije Universiteit Amsterdam, De Boelelaan 1089a, 1081 HV Amsterdam, The Netherlands; 2 Child Health Evaluative Sciences, The Hospital for Sick Children Research Institute, Toronto, ON Canada; 3 IQ Health, Radboud Institute of Health Sciences, Radboud University Medical Center, Nijmegen, The Netherlands; 4 Centre for Patient Reported Outcomes Research, Institute of Applied Health Research, University of Birmingham, Birmingham, UK; 5 Department of General Practice, Erasmus MC, University Medical Center, Rotterdam, The Netherlands; 6 Warwick Applied Health, Warwick Medical School, University of Warwick, Coventry, UK; 7 School of Public Health, University College Cork, Cork, Ireland; 8 Basalt, Leiden/The Hague, The Netherlands; 9 University of Applied Sciences, Utrecht, The Netherlands; 10 Amsterdam UMC, Amsterdam, The Netherlands

**Keywords:** Systematic reviews, Outcome measurement instruments, Measurement properties, Reliability, Validity, COSMIN

## Abstract

**Purpose:**

Systematic reviews evaluating and comparing the measurement properties of outcome measurement instruments (OMIs) play an important role in OMI selection. Earlier overviews of review quality (2007, 2014) evidenced substantial concerns with regards to alignment to scientific standards. This overview aimed to investigate whether the quality of recent systematic reviews of OMIs lives up to the current scientific standards.

**Methods:**

One hundred systematic reviews of OMIs published from June 1, 2021 onwards were randomly selected through a systematic literature search performed on March 17, 2022 in MEDLINE and EMBASE. The quality of systematic reviews was appraised by two independent reviewers. An updated data extraction form was informed by the earlier studies, and results were compared to these earlier studies’ findings.

**Results:**

A quarter of the reviews had an unclear research question or aim, and in 22% of the reviews the search strategy did not match the aim. Half of the reviews had an incomprehensive search strategy, because relevant search terms were not included. In 63% of the reviews (compared to 41% in 2014 and 30% in 2007) a risk of bias assessment was conducted. In 73% of the reviews (some) measurement properties were evaluated (58% in 2014 and 55% in 2007). In 60% of the reviews the data were (partly) synthesized (42% in 2014 and 7% in 2007); evaluation of measurement properties and data syntheses was not conducted separately for subscales in the majority. Certainty assessments of the quality of the total body of evidence were conducted in only 33% of reviews (not assessed in 2014 and 2007). The majority (58%) did not make any recommendations on which OMI (not) to use.

**Conclusion:**

Despite clear improvements in risk of bias assessments, measurement property evaluation and data synthesis, specifying the research question, conducting the search strategy and performing a certainty assessment remain poor. To ensure that systematic reviews of OMIs meet current scientific standards, more consistent conduct and reporting of systematic reviews of OMIs is needed.

**Supplementary Information:**

The online version contains supplementary material available at 10.1007/s11136-024-03706-z.

## Plain English summary

Instruments that measure health outcomes are important for making treatment decisions and understanding diseases. Systematic reviews are used to compare different instruments and help select the best one for a specific situation. Previous studies have shown that the quality of these reviews can vary and may not always meet scientific standards. Since then, new tools and methods have been developed to help systematic review authors in improving the quality of their work. This study looked into the quality of recent systematic reviews of instruments. The study identified important improvements over time. For example, risk of bias is more often evaluated, and the data is analyzed in a better way. However, the study also shows that there are still areas that need improvement. These include formulating a clear research question, and creating a comprehensive search strategy. Ongoing efforts are needed to improve the quality of systematic reviews of instruments. This can be achieved by developing new and accessible resources.

## Introduction

Outcome measurement instruments (OMIs) are used to evaluate the impact of disease and treatment [1–3]. When many different OMIs that measure similar constructs are available [1, 4, 5], the choice for an OMI depends on various aspects, including its quality (i.e., the sufficiency of measurement properties) [6]. Systematic reviews in which the measurement properties of OMIs are critically evaluated and compared are important tools for the selection of an OMI [4], for example in core outcome sets used in research projects or clinical practice [7]. With these systematic reviews, gaps in knowledge about the measurement properties of OMIs can also be identified.

Only well-designed, well-conducted, and comprehensively reported systematic reviews can provide a complete and balanced overview of the measurement properties of OMIs [4]. High-quality systematic reviews have: a well-defined research question; a comprehensive search strategy in multiple databases; independent abstract and full-text article selection; a risk of bias assessment of included studies; a systematic evaluation and syntheses of the results; and a certainty assessment of the body of evidence [8].

Previous overviews appraising the quality of systematic reviews of OMIs identified major limitations in the search strategy, the risk of bias assessment, and the evaluation and synthesis of the measurement properties’ results [9, 10]. These limitations preclude systematic reviews to provide a complete and unbiased overview of the measurement properties of OMIs. This has consequences for knowledge users, who rely on the findings of these systematic reviews and might select suboptimal OMIs to use in their research or clinical practice [11]. This in turn impacts the measurements conducted on patients, which might be invalid and unreliable, and possibly even lead to incorrect healthcare decisions.

**Fig. 1:**
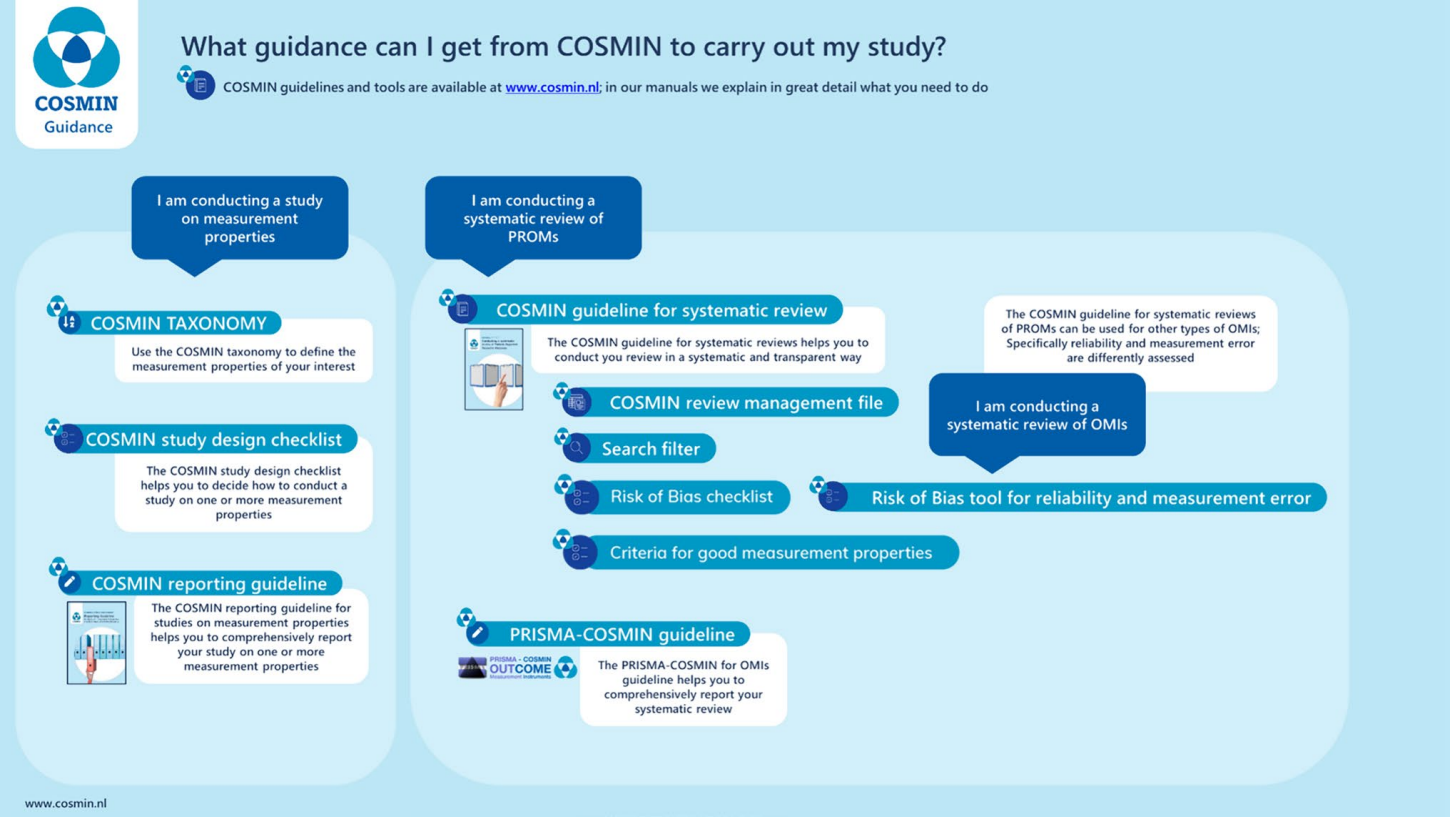
COSMIN tools and methodology

Various methodologies and practical tools have been developed to guide authors in conducting high-quality systematic reviews of OMIs [4, 12, 13]. The methodology and tools developed by the COSMIN (COnsensus-based Standards for the selection of health Measurement INstruments) initiative are the most comprehensive and most widely used (Fig. 1) [14]. Since the most recent overview that assessed the quality of systematic reviews of OMIs, published in 2016 [10], the COSMIN guideline for systematic reviews has been developed [4] and the COSMIN risk of bias checklist has been updated [15, 16]. Other methodologies and tools for critical appraisal of OMIs have also been developed and updated since then [12, 17]. When reading or reviewing such systematic reviews, even those that claim to have used these guidelines, we often observe flaws in the design, conduct, and reporting. The aim of this overview of reviews was therefore to investigate whether the quality of recent systematic reviews of OMIs lives up to the current scientific standards. As a secondary aim, we explored which aspects have notably improved over time.

## Methods

The study protocol was registered in the PROSPERO database, number CRD42022320675 [18]. There were no important deviations from the protocol. The study was reported according to the preferred reporting items for overviews of reviews (PRIOR) statement [19]. Consistent with the previous overview [10], we randomly selected 100 out of 136 most recent systematic reviews from the COSMIN database of systematic reviews [20]. These reviews were identified while updating the COSMIN database through a systematic literature search performed on March 17, 2022 in MEDLINE (through PubMed) and EMBASE (through www.embase.com), and concerned systematic reviews of OMIs published from June 1, 2021 onwards. The search strategy consisted of search terms for systematic reviews, search terms for OMIs, and a validated search filter for measurement properties [21]. The full search strategy can be found in Supplementary File 1. Table 1 contains inclusion and exclusion criteria for the COSMIN database [20]. We defined systematic reviews of OMIs as peer-reviewed studies with a systematic search in at least one electronic database which aimed to summarize evidence on the measurement properties of all OMIs of interest to the review.

**Table 1:** Inclusion and exclusion criteria for the COSMIN database [20]

Inclusion criteria	Exclusion criteria
Search performed in at least one electronic database	It concerns a prognostic review (i.e. aiming to predict an outcome using multivariable analyses)
Aim is to identify all OMIs of interest and summarize evidence on their measurement properties	Aim is to evaluate one or the most commonly used OMIs
Construct of interest is (aspect of) health status, based on Wilson & Cleary model [22]: Biological and physiological processes Symptoms Physical functioning Social/psychological functioning General health perceptions Health-related quality of life	Reviews that include only randomized controlled trials
Study population is humans (patients or general population)	Non-English reviews
Instrument of interest is OMIs, i.e., instruments which can be applied in longitudinal studies to monitor changes in health over time	Instrument is a diagnostic or screening instrument
At least one measurement property of the included OMIs is evaluated and reported	

Eligibility for inclusion in the COSMIN database was determined by one reviewer (IS). All reviewers confirmed that each review appraised in the current study complied with the inclusion and exclusion criteria. If a review was selected from the COSMIN database that should have been excluded (false-positive), this review was replaced by a randomly selected new review after confirming exclusion by a third reviewer (LM).

A study-specific data extraction form (Supplementary File 2) was developed to appraise the quality of systematic reviews of OMIs, which includes both methodological quality and reporting quality—two aspects that cannot be considered separately when appraising the quality of published OMI systematic reviews. The data extraction form was based on criteria used in previous studies [9, 10], which were updated for this study. The data extraction form contained items on the key elements of the review (i.e., construct, population, type of OMI, and measurement properties of interest), search strategy, eligibility criteria, article selection, data extraction, risk of bias assessment, evaluation of measurement properties, data synthesis, certainty assessment, presentation of results, instrument recommendation, and elements of open science). Specifically, the appropriateness of the search for the construct, population, type of OMI and measurement properties was based on published search filters [21, 23], search terms found at blocks.bmi-online.nl, and the reviewers own knowledge. For each item, two independent reviewers extracted information on whether this was done/reported in the included reviews. No attempts were made to verify information with study authors. Reviewers also noted any major methodological and reporting flaws for each of these aspects.

The data extraction form was pilot tested with six OMI systematic reviews [24–29] by two independent reviewers (different pairs of EE, CT, and LM). A subsequent update was done after training the other reviewers, who were instructed to extract data for one of these six reviews [25]. Discrepancies were discussed during two 90-min Zoom meetings intended to standardize the data extraction process. After these meetings, the data extraction form and instructions on how to appraise each systematic review were finalized, and five pairs of reviewers were formed (EE&JP/IS, LM&DO, CT&IA, KH&KM, AC&OA). Each reviewer pair subsequently appraised the quality of 18–19 systematic reviews independently. Reviews were not appraised by a reviewer who was a co-author or had a potential conflict of interest. Discrepancies between the pair of reviewers were resolved through discussion. Appraisals of reviewers were descriptively synthesized by review counts and a qualitative comparison of the results was made to the results of previous studies [9, 10], if possible.

## Results

Characteristics of the 100 systematic reviews are presented in Table 2. Half of the included reviews focused on patient-reported outcomes, 30% focused on non-patient-reported outcomes, and 20% on a combination of both. The aspect of health of the construct of interest in the reviews was mostly functional status (62%), symptom status (56%), and/or general health perceptions (36%). Reviews focused on a variety of populations, such as children and (older) adults with a variety of diseases and conditions. Questionnaires (77%), clinical rating scales (41%), and/or performance-based tests (24%) were the OMI types most often included.

**Table 2:** Characteristics of systematic reviews of outcome measurement instruments (n = 100)

Characteristic	Reviews (n)
Construct of interest	
Patient-reported outcome	51
Non-patient-reported outcome	29
Patient- and non-patient-reported outcome	20
Level of health of the construct of interest*	
Biological and physiological variables	29
Symptom status	56
* Physical state*	42
* Emotional state*	19
* Cognitive/mental state*	13
Functional status	63
* Physical functioning*	48
* Social functioning*	22
* Cognitive/mental functioning*	21
* Role functioning*	9
General health perceptions	37
* Health-related quality of life*	36
* Self-rated health*	3
Overall quality of life	9
Age of the population of interest	
Children 0–18	20
Adults 18 +	58
Both	22
Condition of the population of interest*	
Circulatory system	11
Congenital and genetic conditions	8
Digestive, endocrine and metabolic system	16
Ear, eye, and respiratory system	14
Genitourinary and reproductive system	9
Infections and parasites	5
Injuries and external causes	13
Mental and behavioral health	19
Musculoskeletal system	30
Neoplasms	15
Nervous system	16
Perinatal and pediatric health	4
Skin and subcutaneous tissue	10
Factors/symptoms influencing health status/contact with health services	42
OMI type included*	
Questionnaires	41
Clinical rating scales	41
Performance-based tests	24
Observations	7
Interviews	7
Imaging tests	6
Laboratory tests	6
Diaries	5
Videos	1
Measurement properties considered per review, median [range]*	[1–9]
Content validity	64
Structural validity	46
Internal consistency	69
Cross-cultural validity/measurement invariance	37
Reliability	87
Measurement error	47
Construct validity	82
Criterion validity	50
Responsiveness	62

^*^Multiple characteristics could be reported for the same review

Syntheses of the quality appraisal of the 100 systematic reviews of OMIs [24–123] are presented in Table 3. Supplementary File 2 contains the completed data extraction form, whereas Supplementary File 3 contains the data from Table 3 in comparison with the results of the two previous studies [9, 10].

**Table 3:** Quality appraisal of systematic reviews of outcome measurement instruments

Quality aspect	% Reviews (n = 100)
**Key elements**	
Key elements included in title	
Construct	80
Population	82
Type of OMI	66
Measurement properties	34
Systematic review	80
Key elements included in aim	
Construct	87
Population	81
Type of OMI	76
Measurement properties	76
**Search strategy**	
Search strategy matched aim	78
Search syntax for at least 1 database provided	70
Search appropriate for Construct	
Yes	49
Unclear	31
No	20
Population	
Yes	59
Unclear	21
No	20
Type of OMI	
Yes	48
Unclear	12
No	40
Measurement properties	
Yes	56
Unclear	10
No	34
Number of databases searched, median [range]	[1–14]
MEDLINE	98
EMBASE	56
Reference checking used	66
No time limits used in search or arguments provided for used of time limits	77
No language restrictions used in search	66
No other notable restrictions used in search	77
**Eligibility criteria**	
Inclusion and exclusion criteria clearly defined	75
Eligibility criteria matched aim	83
No other notably criteria used in eligibility	58
**Article selection**	
Abstract selection by at least 2 independent reviewers	
Yes	62
Partly	3
Unclear	26
No	9
Full-text selection by at least 2 independent reviewers	
Yes	67
Partly	2
Unclear	27
No	4
**Data extraction**	
Data extraction by at least 2 independent reviewers	
Yes	39
Partly	3
Unclear	44
No	14
**Risk of bias assessment**	
Methodological quality assessment of included studies	63
Methodological quality assessment by at least 2 independent reviewers	
Yes	62
Partly	1
Unclear	33
No	3
**Measurement property evaluation**	
Quality of the OMI (measurement properties) evaluated	
Yes	59
Some measurement properties	14
No	27
Criteria for measurement properties specified	
Yes	67
For some measurement properties	14
No	19
Evaluation of each subscale (if multidimensional)	
Yes	18
Partly	5
Unclear	26
No	51
Measurement properties evaluated by at least 2 independent reviewers	
Yes	21
Partly	1
Unclear	70
No	8
**Data synthesis**	
Data synthesis performed (if possible)	
Yes	31
Partly	57
Unclear	3
No	8
Data synthesis performed for each subscale (if multidimensional)	
Yes	13
Unclear	50
No	37
Data synthesis methods clearly described	47
Data synthesis performed at the level of	
Measurement properties	84
Only domains of measurement properties	13
Only subscales or instruments	4
Data synthesis performed by at least 2 independent reviewers	
Yes	18
Unclear	75
No	7
**Certainty assessment**	
Quality of the evidence graded	33
Quality of the evidence graded for each subscale (if multidimensional)	
Yes	15
Unclear	19
No	67
Quality of the evidence graded by at least 2 independent reviewers	
Yes	27
Unclear	70
No	3
**Presentation of results**	
Flow chart provided	96
Reasons for excluding full text articles reported	
Full information (numbers for each reason)	65
Some information (reasons, but not specifying numbers)	20
No	15
Included instruments in accordance with inclusion criteria	
Yes	86
Unclear	12
No	2
Results of measurement properties reported as raw data	
Yes	42
For some measurement properties	30
No	28
**Instrument recommendation**	
Recommendations for instruments made	42
Recommendations made for each construct of interest	25
OMI recommendation consistent with evidence appraisal	
Yes	55
Partly	7
Unclear	24
No	14

### Key elements

Only 11% of the reviews had a title that included all four key elements (i.e., construct, population, type of OMI, and measurement properties of interest) and the fact that it concerned a systematic review. In titles of the remaining reviews, often no reference to measurement property evaluation was made. 47% of the reviews had a title that omitted at least 2 key elements and/or the fact that it concerned a systematic review. The term ‘scoping review’ was used in 7% of the reviews. In 45% of the reviews all 4 key elements were included in the aim, whereas in 18% of the review aims at least 2 key elements were not reported. Major flaws identified in the aim were often that the aim was unclear or vague, for example by stating that the aim was “to discuss validity” [121], or “to provide information about frailty instruments” [94].

### Search strategy

In 78% of the reviews the search strategy matched the research aim. When there was a mismatch between the aim and the search strategy, often the aim was to identify all available OMIs, whereas search terms for measurement properties were included. Hence, only OMIs with evidence for the measurement properties were identified.

Only 27% of the reviews had an appropriate search strategy with respect to search terms used for both the construct, population, OMI type and measurement properties. Search terms for OMI type were not appropriate for 40% of the reviews because relevant synonyms or search terms were not included. Search terms for measurement properties were deemed inappropriate for 34% of the reviews.

The number of databases searched ranged from 1–14, with a median of 4. MEDLINE was searched in 98% of the included reviews, whereas EMBASE was searched in 56%. Only 66% of the reviews performed reference checking of included articles.

### Eligibility criteria and article selection

In 75% of the reviews the eligibility criteria were clearly defined, and in 83% the eligibility criteria matched the research aim. Mismatches often concerned that the aim was to identify all available or used OMIs, whereas eligibility criteria included that the study should report on measurement properties, hence resulting in including only OMIs that were validated to at least some extent. In 42% of the reviews other notable eligibility criteria were used, such as only including OMIs that were reported in at least a certain number of articles, only including validation studies of original OMIs or certain (language) versions, excluding studies of low quality, or excluding OMIs that were described in previously published systematic reviews.

In 65% and 69% of the reviews, respectively abstract and full text selection was (partly) done by at least 2 independent reviewers, compared to 41% and 38% in 2014. Data extraction was (partly) done by at least 2 independent reviewers in 42%, compared to 25% in both 2014 and 2007. In most other cases it was unclear whether two independent reviewers were involved.

### Risk of bias assessment

The methodological quality (i.e., risk of bias) of the studies was evaluated in 63% of the reviews, compared to 41% in 2014 and 30% in 2007. In 62% of those reviews, the quality assessment was done by at least two reviewers independently. For 33% of the reviews this was unclear.

### Measurement property evaluation

In 73% of the reviews (some) measurement properties of the included OMIs were evaluated, compared to 58% in 2014 and 55% in 2007. This means that in these reviews a judgement was made about the sufficiency of the measurement properties, rather than providing only the results of measurement properties. For those reviews in which (some) measurement properties were evaluated, (a reference to) criteria for measurement properties were provided in 81% of the reviews; in 19% of the reviews it was not clear on what criteria judgements were based. In those reviews in which measurement properties were evaluated and that included multidimensional OMIs, only 18% evaluated each subscale separately. In 22% of the reviews the evaluation of measurement properties was (partly) done by at least two independent reviewers.

### Data synthesis and certainty assessment

Data synthesis, in which results from multiple studies on the same OMI were combined, was (partly) performed in 60% of the reviews, compared to 44% in 2014 and 7% in 2007. In those reviews in which data synthesis was performed and that included multidimensional OMIs, synthesis was performed for each subscale separately in only 13% of the cases. Methods for data syntheses were clearly described in 47% of the reviews. In 84% of the reviews data synthesis was performed for each measurement property separately. Data synthesis was performed by at least 2 independent reviewers for 18% of the reviews.

In 33% of the reviews, a certainty assessment was done in which the quality of the evidence was graded. Quality of the evidence was graded by at least 2 independent reviewers in 27% of the reviews.

### Results and instrument recommendation

A flowchart was provided in 96% of the reviews, often with reasons for excluding full texts (85% vs. 55% in 2014). Included instruments were in 86% of the reviews in accordance with the inclusion criteria. In 72% of the reviews, the results of (some) measurement properties were reported as raw data.

In almost half of the reviews (42%) recommendations on which instrument (not) to use were made. In 25% of the reviews, recommendations were made for each construct of interest. In 62% of the reviews the recommendations made were consistent with the evidence appraisal.

A summary of the main results with recommendations for future OMI systematic reviews is provided in Table 4.

**Table 4:** Overview of main findings and recommendations for future OMI systematic reviews

Features of high-quality systematic reviews of outcome measurement instruments		Main findings	Recommendations
	Well-defined research question/aim	Over half of the reviews had an unclear research question or aim	Formulate a research question/aim based on the four key elements: construct, population, (type of) instrument, measurement properties
	Comprehensive search strategy	Three-quarters of the reviews had an inappropriate or incomprehensive search strategy	If search terms for (type of) instrument and measurement properties are needed, use validated search filters (e.g., [21, 23])
	Independent abstract and full-text selection	For one-third of the reviews it was unclear whether article selection was done independently	Independently select articles and report how article selection took place
	Risk of bias assessment of included studies	One-third of the reviews had no risk of bias assessment of included studies	Conduct a risk of bias assessment using an appropriate tool, for example the COSMIN risk of bias checklist [16] or tool [15]
	Measurement property evaluation	In a quarter of the reviews no measurement properties were evaluated	Evaluate the measurement properties of included instruments using established criteria, for example criteria for good measurement properties [4]
	Synthesis of results, including instrument recommendations	In almost half of the reviews evidence from multiple studies for the same measurement property and instrument were not synthesized; recommendations on what instrument (not) to use were made in less than half of the reviews	Synthesize study results on the same measurement property of the same instrument and make recommendations on what instrument (not) to use
	Certainty assessment of the body of evidence	Two-thirds of the review did not include a certainty assessment of the body of evidence	Make a certainty assessment of the body of evidence using for example the (modified) GRADE system [124]

## Discussion

This overview of reviews aimed to investigate whether the quality of recent systematic reviews of OMIs lives up to the current scientific standards and which aspects have notably improved over time. Compared to previous studies [9, 10], we found marked improvements in the conduct of risk of bias assessments, evaluation of measurement properties, and performance of formal data syntheses. Despite this, further improvements in these areas are necessary, as well as with respect to the research question and search strategy.

Over half of the reviews included in this study had an unclear research question or aim, for example with respect to the population of interest, the measurement properties that were evaluated, or the type of OMIs that were included. Including the four key elements, analogue to the PICO (population, intervention, comparison, outcome) format in systematic reviews of interventions [4, 8, 125], helps to formulate a well-defined research question and facilitates the development of an appropriate search strategy. Without a clear research question, it is not possible to assess the comprehensiveness of the search strategy.

Almost three-quarters of the reviews had an inappropriate or incomprehensive search strategy, often because inappropriate search terms for OMI type or measurement properties were included. It is preferred not to use search terms for OMI type to avoid missing any studies; however, if search terms are needed because of too many results, a search filter exists for PROMs [23]. A highly sensitive search filter also exists for measurement properties [21], but it was used in only 14 reviews. While searching both MEDLINE and EMBASE is recommended as a minimum by Cochrane [126], almost half of the reviews included in this study did not search EMBASE. Similarly, whilst reference checking is recommended [126], this was not reported by a third of the reviews. Through reference checking, one can also confirm the comprehensiveness of the search strategy: if many relevant articles were found through reference checking, the search was probably not comprehensive and important studies may have been missed [126].

In almost half of the reviews poorly justified eligibility criteria were used, e.g., only including OMIs in a certain language, or excluding OMIs that were included in previous systematic reviews. Such unintuitive eligibility criteria might negatively impact the inclusion of relevant studies or OMIs, hampering a complete synthesis of the body of available evidence. The number of reviews in which article selection and data extraction was conducted by at least 2 independent reviewers increased compared to previous overviews [9, 10].

Whilst a marked increase in the number of reviews that included a risk of bias assessment was found (63% currently compared to 41% in 2014 and 30% in 2007 [9, 10]), opportunities for improvement remain. Evaluating risk of bias in empirical studies on measurement properties is important, because results might not be valid if a study has bias. For example, relevant items might be missing in a PROM if patients were not involved in its development, or the reliability of an OMI might be underestimated if the time interval between test and retest is (too) long. The COSMIN risk of bias checklist [16] or tool [15] were specifically developed for this purpose and were used in 47 reviews. Other risk of bias tools reported in the reviews [43, 70, 82, 101, 120] included, for example, the QUADAS-2 [127], QAREL [128], ROBINS-I [129], and Newcastle–Ottawa quality assessment scale [130]. These tools are, however, not specifically developed to assess the methodological quality of empirical measurement property studies and may not identify important bias.

The number of reviews in which measurement properties were formally evaluated has notably increased since 2007 (73% currently compared to 58% in 2014 and 55% in 2007 [9, 10]). In 14 reviews, however, it was not clear which criteria were used. In several reviews, authors mistakenly used risk of bias or certainty assessment ratings as a measure of OMI quality. However, these ratings refer to the quality of the study and the quality of the evidence, respectively, and not to the quality of the OMI (i.e., its measurement properties).

A clear increase in the number of reviews in which a data synthesis was performed was also observed (60% currently compared to 42% in 2014 and 7% in 2007 [9, 10]). However, the methods for data synthesis were often unclearly described and only in a third of the reviews a certainty assessment of the body of evidence was conducted. Potentially, the publication of the COSMIN guideline for systematic reviews of PROMs [4] in 2018 increased the number of reviews in which a data synthesis was performed. This guideline details how to synthesize multiple studies on the same measurement property for the same OMI, although more guidance might be necessary.

Each subscale in a multidimensional instrument should be considered a separate instrument as it represents a unique construct with measurement properties often varying between subscales [4]. However, we observed that few studies separately evaluated measurement properties or conducted an evidence synthesis at the subscale level. By not evaluating each subscale separately, a review therefore presents an incomplete picture of the measurement properties for the given scale.

Less than half of the reviews made recommendations about which OMI (not) to use. The conclusions of systematic reviews will be used by other researchers and clinicians who need to select an OMI for their purpose, although the selection of the most appropriate OMI may depend on the context and situation. Clear, evidence-based recommendations on which OMI (not) to use will help others in their OMI selection and contribute to the standardization of OMIs.

Although two-thirds of the reviews purport to include an evaluation of content validity, there is doubt over the thoroughness of these evaluations. Whilst 25 reviews reported application of the COSMIN guideline for evaluating content validity, only 13 appear to have applied it correctly. One of the steps in the assessment of content validity according to the COSMIN guideline is the evaluation of the content by reviewers themselves. This step was often lacking. Other flaws included not distinguishing between development and content validity studies, and only conducting a risk of bias assessment without evaluating the content validity of the OMI.

Other major flaws that we observed in some reviews were confusing the quality of the study (i.e., risk of bias) with the quality of the OMI (i.e., its measurement properties) or making recommendations based on certainty assessment rather than the sufficiency of measurement properties.

### Towards high quality OMI systematic reviews

Systematic reviews of OMIs are difficult to conduct, and this study shows that the availability of methodology and tools that guide authors in the conduct of their systematic review does not translate automatically into high-quality systematic reviews. Besides more and better resources, behavioral change techniques [131], implementation strategies, and knowledge translation activities are needed to improve systematic review quality. Several of these have recently been developed or are being considered. First, the COSMIN guideline for systematic reviews has recently been updated and made more user-friendly to better facilitate reviewers [132]. Second, a newly developed animated video explains the key steps of conducting a systematic review of OMIs (available at https://www.cosmin.nl/). Third, a reporting guideline for OMI systematic reviews has recently been developed [133], and knowledge translation activities have been implemented to increase its uptake. Last, a course on how to conduct OMI systematic reviews is being developed to educate reviewers more thoroughly. To alert systematic review authors to the various tools available, an automated email can be sent to authors registering their review in PROSPERO. PROSPERO is a database for registering systematic reviews of health related outcomes [134], and although less than half of the included reviews reported prospective registration, such an email alert might increase the uptake of tools and improve the quality of future OMI systematic reviews.

### Limitations

An important limitation of the current study is the potential subjectivity in appraising the quality of systematic reviews. We attempted to use a rigorous and standardized data extraction process, in which we pilot tested and improved the data extraction form, provided training to reviewers who were already experts in systematic reviews of OMIs, and assigned systematic reviews to reviewer pairs who independently appraised their quality and reached consensus about any discrepancies. However, because of large variations in the systematic reviews included, some degree and variation of subjective judgement in appraising the quality of systematic reviews could not be avoided. Second, some of the included reviews might not have been systematic reviews by definition, as the inclusion criteria were not stringent in that respect. We decided to include a review if at least one measurement property was evaluated (i.e., some degree of judgement was made about the sufficiency of a measurement property, as opposed to only providing an overview of the measurement properties). Third, we were unable to compare all quality aspects historically, because not all aspects were rated in the studies conducted in 2014 and 2007 [9, 10]. Compared to the previous studies, the current appraisal is the most comprehensive, and new elements were added, such as inclusion of key elements in the title, specification of criteria for measurement properties, evaluation of subscales, and assessment of certainty. Fourth, we randomly selected 100 recent reviews that fulfilled the eligibility criteria, out of a set of 136 reviews that were identified while updating the COSMIN database [20]. Our aim was not to include all available systematic reviews but rather to appraise and compare the quality of a random sample of the most recently published reviews with a set of reviews published respectively 8 and 15 years ago. We believe that the inclusion of additional reviews would not have altered our findings. Lastly, the appraisal of the reviews’ quality was hampered by poor reporting, for example with respect to the process of data synthesis or the number of independent reviewers involved in each of the steps of the review process. The recently developed PRISMA-COSMIN for OMIs reporting guideline could improve the reporting of OMI systematic reviews [133]. Although the current study is not a one-to-one baseline assessment of reporting aspects required by PRISMA-COSMIN for OMIs, most reporting items have been included in the current quality appraisal. Because our aim was to assess whether the quality of recent systematic reviews lived up to the current scientific standards, including reporting quality, we have not contacted the authors of the included systematic reviews to provide additional information.

## Conclusion

In conclusion, this overview of 100 reviews published after June 2021 found, compared to previous overviews of reviews, a clear improvement in the number of OMI systematic reviews that conducted a risk of bias assessment, evaluated the measurement properties of included OMIs, and conducted a data synthesis. However, room for improvement in these areas remains. Improvements regarding the research question and search strategy are urgently needed, as more than half of the reviews likely missed important studies. To ensure that systematic reviews of OMIs meet current scientific standards, more consistent conduct and reporting of systematic reviews of OMIs is needed.

## Supplementary Information

Below is the link to the electronic supplementary material.

Supplementary material 1 (DOCX 22 kb)

Supplementary material 2 (XLSX 90 kb)

Supplementary material 3 (DOCX 17 kb)

## Data Availability

All data supporting the findings of this study are available within the paper and its Supplementary Information.
