# Supplementary material for: Methodological quality of 100 recent systematic reviews of health-related outcome measurement instruments: an overview of reviews
Source: Qual Life Res. 2024 Jul 3;33(10):2593–609. doi: 10.1007/s11136-024-03706-z (PMC11452433; doi:10.1007/s11136-024-03706-z)
Supplement: Supplementary file 1 — Supplementary material 1 (DOCX 22 kb) [file 11136_2024_3706_MOESM1_ESM.docx]

**Supplementary File** **1.** Search strategy for finding systematic reviews of outcome measurement instruments

**PubMed (March 17, 2022)**

#1: (instruments[tiab] OR scales[tiab] OR Questionnaires[tiab] OR measures[ti] OR methods[ti] OR outcome measurements[tiab] OR (tests[tiab] AND review[tiab]) OR Questionnaires[MeSH] OR interview[MeSH])

#2: (systematic[sb] OR (literature AND search*) OR (Medline AND search*) OR review[ti])

#3: (instrumentation[sh] OR methods[sh] OR “Validation Studies”[pt] OR “Comparative Study”[pt] OR “psychometrics”[MeSH] OR psychometr*[tiab] OR clinimetr*[tw] OR clinometr*[tw] OR “outcome assessment (health care)”[MeSH] OR “outcome assessment”[tiab] OR “outcome measure*”[tw] OR “observer variation”[MeSH] OR “observer variation”[tiab] OR “Health Status Indicators”[Mesh] OR “reproducibility of results”[MeSH] OR reproducib*[tiab] OR “discriminant analysis”[MeSH] OR reliab*[tiab] OR unreliab*[tiab] OR valid*[tiab] OR “coefficient of variation”[tiab] OR coefficient[tiab] OR homogeneity[tiab] OR homogeneous[tiab] OR “internal consistency”[tiab] OR (cronbach*[tiab] AND (alpha[tiab] OR alphas[tiab])) OR (item[tiab] AND (correlation*[tiab] OR selection*[tiab] OR reduction*[tiab])) OR agreement[tw] OR precision[tw] OR imprecision[tw] OR “precise values”[tw] OR test-retest[tiab] OR (test[tiab] AND retest[tiab]) OR (reliab*[tiab] AND (test[tiab] OR retest[tiab])) OR stability[tiab] OR interrater[tiab] OR inter-rater[tiab] OR intrarater[tiab] OR intra-rater[tiab] OR intertester[tiab] OR inter-tester[tiab] OR intratester[tiab] OR intra-tester[tiab] OR interobserver[tiab] OR inter-observer[tiab] OR intraobserver[tiab] OR intra-observer[tiab] OR intertechnician[tiab] OR inter-technician[tiab] OR intratechnician[tiab] OR intra-technician[tiab] OR interexaminer[tiab] OR inter-examiner[tiab] OR intraexaminer[tiab] OR intra-examiner[tiab] OR interassay[tiab] OR inter-assay[tiab] OR intraassay[tiab] OR intra-assay[tiab] OR interindividual[tiab] OR inter-individual[tiab] OR intraindividual[tiab] OR intra-individual[tiab] OR interparticipant[tiab] OR inter-participant[tiab] OR intraparticipant[tiab] OR intra-participant[tiab] OR kappa[tiab] OR kappa’s[tiab] OR kappas[tiab] OR repeatab*[tw] OR ((replicab*[tw] OR repeated[tw]) AND (measure[tw] OR measures[tw] OR findings[tw] OR result[tw] OR results[tw] OR test[tw] OR tests[tw])) OR generaliza*[tiab] OR generalisa*[tiab] OR concordance[tiab] OR (intraclass[tiab] AND correlation*[tiab]) OR discriminative[tiab] OR “known group”[tiab] OR “factor analysis”[tiab] OR “factor analyses”[tiab] OR “factor structure”[tiab] OR “factor structures”[tiab] OR dimension*[tiab] OR subscale*[tiab] OR (multitrait[tiab] AND scaling[tiab] AND (analysis[tiab] OR analyses[tiab])) OR “item discriminant”[tiab] OR “interscale correlation*”[tiab] OR error[tiab] OR errors[tiab] OR “individual variability”[tiab] OR “interval variability”[tiab] OR “rate variability”[tiab] OR (variability[tiab] AND (analysis[tiab] OR values[tiab])) OR (uncertainty[tiab] AND (measurement[tiab] OR measuring[tiab])) OR “standard error of measurement”[tiab] OR sensitiv*[tiab] OR responsive*[tiab] OR (limit[tiab] AND detection[tiab]) OR “minimal detectable concentration”[tiab] OR interpretab*[tiab] OR ((minimal[tiab] OR minimally[tiab] OR clinical[tiab] OR clinically[tiab]) AND (important[tiab] OR significant[tiab] OR detectable[tiab]) AND (change[tiab] OR difference[tiab])) OR (small*[tiab] AND (real[tiab] OR detectable[tiab]) AND (change[tiab] OR difference[tiab])) OR “meaningful change”[tiab] OR “ceiling effect”[tiab] OR “floor effect”[tiab] OR “Item response model”[tiab] OR IRT[tiab] OR Rasch[tiab] OR “Differential item functioning”[tiab] OR DIF[tiab] OR “computer adaptive testing”[tiab] OR “item bank”[tiab] OR “cross-cultural equivalence”[tiab])

#4: (‘delphi-technique’[ti] OR cross-sectional[ti] OR "addresses"[Publication Type] OR "biography"[Publication Type] OR "case reports"[Publication Type] OR "comment"[Publication Type] OR "directory"[Publication Type] OR "editorial"[Publication Type] OR "festschrift"[Publication Type] OR "interview"[Publication Type] OR "lectures"[Publication Type] OR "legal cases"[Publication Type] OR "legislation"[Publication Type] OR "letter"[Publication Type] OR "news"[Publication Type] OR "newspaper article"[Publication Type] OR "patient education handout"[Publication Type] OR "popular works"[Publication Type] OR "congresses"[Publication Type] OR "consensus development conference"[Publication Type] OR "consensus development conference, nih"[Publication Type] OR "practice guideline"[Publication Type]) NOT ("animals"[MeSH Terms] NOT "humans"[MeSH Terms])

#1 AND #2 AND #3 NOT #4 AND (2021/6/1:3000/12/12[pdat])

**EMBASE (March 17, 2022)**

#1: instruments:ti,ab OR scales:ti,ab OR questionnaires:ti,ab OR measures:ti OR methods:ti OR outcome-measurements:ti,ab OR (tests:ti,ab AND review:ti,ab) OR 'outcomes research'/de OR 'treatment outcome'/de OR 'psychologic test'/de OR 'measurement'/de OR 'functional assessment'/de OR 'pain assessment'/de OR 'questionnaire'/de OR 'rating scale'/de

#2: review:ti OR (literature AND search*) OR (medline AND search*) OR 'systematic review'/exp

#3: ‘intermethod comparison’/exp OR ‘data collection method’/exp OR ‘validation study’/exp OR ‘feasibility study’/exp OR ‘pilot study’/exp OR ‘psychometry’/exp OR ‘reproducibility’/exp OR reproducib*:ab,ti OR ‘audit’:ab,ti OR psychometr*:ab,ti OR clinimetr*:ab,ti OR clinometr*:ab,ti OR ‘observer variation’/exp OR ‘observer variation’:ab,ti OR ‘discriminant analysis’/exp OR ‘validity’/exp OR reliab*:ab,ti OR valid*:ab,ti OR ‘coefficient’:ab,ti OR ‘internal consistency’:ab,ti OR (cronbach*:ab,ti AND (‘alpha’:ab,ti OR ‘alphas’:ab,ti)) OR ‘item correlation’:ab,ti OR ‘item correlations’:ab,ti OR ‘item selection’:ab,ti OR ‘item selections’:ab,ti OR ‘item reduction’:ab,ti OR ‘item reductions’:ab,ti OR ‘agreement’:ab,ti OR ‘precision’:ab,ti OR ‘imprecision’:ab,ti OR ‘precise values’:ab,ti OR ‘test-retest’:ab,ti OR (‘test’:ab,ti AND ‘retest’:ab,ti) OR (reliab*:ab,ti AND (‘test’:ab,ti OR ‘retest’:ab,ti)) OR ‘stability’:ab,ti OR ‘interrater’:ab,ti OR ‘inter-rater’:ab,ti OR ‘intrarater’:ab,ti OR ‘intra-rater’:ab,ti OR ‘intertester’:ab,ti OR ‘inter-tester’:ab,ti OR ‘intratester’:ab,ti OR ‘intratester’:ab,ti OR ‘interobeserver’:ab,ti OR ‘inter-observer’:ab,ti OR ‘intraobserver’:ab,ti OR ‘intraobserver’:ab,ti OR ‘intertechnician’:ab,ti OR ‘inter-technician’:ab,ti OR ‘intratechnician’:ab,ti OR ‘intratechnician’:ab,ti OR ‘interexaminer’:ab,ti OR ‘inter-examiner’:ab,ti OR ‘intraexaminer’:ab,ti OR ‘intraexaminer’:ab,ti OR ‘interassay’:ab,ti OR ‘inter-assay’:ab,ti OR ‘intraassay’:ab,ti OR ‘intra-assay’:ab,ti OR ‘interindividual’:ab,ti OR ‘inter-individual’:ab,ti OR ‘intraindividual’:ab,ti OR ‘intra-individual’:ab,ti OR ‘interparticipant’:ab,ti OR ‘inter-participant’:ab,ti OR ‘intraparticipant’:ab,ti OR ‘intraparticipant’:ab,ti OR ‘kappa’:ab,ti OR ‘kappas’:ab,ti OR ‘coefficient of variation’:ab,ti OR repeatab*:ab,ti OR (replicab*:ab,ti OR ‘repeated’:ab,ti AND (‘measure’:ab,ti OR ‘measures’:ab,ti OR ‘findings’:ab,ti OR ‘result’:ab,ti OR ‘results’:ab,ti OR ‘test’:ab,ti OR ‘tests’:ab,ti)) OR generaliza*:ab,ti OR generalisa*:ab,ti OR ‘concordance’:ab,ti OR (‘intraclass’:ab,ti AND correlation*:ab,ti) OR ‘discriminative’:ab,ti OR ‘known group’:ab,ti OR ‘factor analysis’:ab,ti OR ‘factor analyses’:ab,ti OR ‘factor structure’:ab,ti OR ‘factor structures’:ab,ti OR ‘dimensionality’:ab,ti OR subscale*:ab,ti OR ‘multitrait scaling analysis’:ab,ti OR ‘multitrait scaling analyses’:ab,ti OR ‘item discriminant’:ab,ti OR ‘interscale correlation’:ab,ti OR ‘interscale correlations’:ab,ti OR (‘error’:ab,ti OR ‘errors’:ab,ti AND (measure*:ab,ti OR correlat*:ab,ti OR evaluat*:ab,ti OR ‘accuracy’:ab,ti OR ‘accurate’:ab,ti OR ‘precision’:ab,ti OR ‘mean’:ab,ti)) OR ‘individual variability’:ab,ti OR ‘interval variability’:ab,ti OR ‘rate variability’:ab,ti OR ‘variability analysis’:ab,ti OR (‘uncertainty’:ab,ti AND (‘measurement’:ab,ti OR ‘measuring’:ab,ti)) OR ‘standard error of measurement’:ab,ti OR sensitiv*:ab,ti OR responsive*:ab,ti OR (‘limit’:ab,ti AND ‘detection’:ab,ti) OR ‘minimal detectable concentration’:ab,ti OR interpretab*:ab,ti OR (small*:ab,ti AND (‘real’:ab,ti OR ‘detectable’:ab,ti) AND (‘change’:ab,ti OR ‘difference’:ab,ti)) OR ‘meaningful change’:ab,ti OR ‘minimal important change’:ab,ti OR ‘minimal important difference’:ab,ti OR ‘minimally important change’:ab,ti OR ‘minimally important difference’:ab,ti OR ‘minimal detectable change’:ab,ti OR ‘minimal detectable difference’:ab,ti OR ‘minimally detectable change’:ab,ti OR ‘minimally detectable difference’:ab,ti OR ‘minimal real change’:ab,ti OR ‘minimal real difference’:ab,ti OR ‘minimally real change’:ab,ti OR ‘minimally real difference’:ab,ti OR ‘ceiling effect’:ab,ti OR ‘floor effect’:ab,ti OR ‘item response model’:ab,ti OR ‘irt’:ab,ti OR ‘rasch’:ab,ti OR ‘differential item functioning’:ab,ti OR ‘dif’:ab,ti OR ‘computer adaptive testing’:ab,ti OR ‘item bank’:ab,ti OR ‘cross-cultural equivalence’:ab,ti#4: 'Delphi technique':ti OR Cross-sectional:ti OR 'case report'/de OR letter:it OR animal/exp OR 'animal model'/exp OR 'animal experiment'/exp

#5: #1 AND #2 AND #3;

#6: #5 NOT #4

#7: #6 AND [1-6-2021]/sd

**Supplementary File 2.** Completed data extraction form

[see Excel document]

**Supplementary File 3.** Quality appraisal of systematic reviews of outcome measurement instruments and comparisons to previous reviews [9, 10]

| Quality aspect | % this study (n=100) | % 2014 study  (n=102)* | % 2007 study (n=148)* |
| --- | --- | --- | --- |
| ***Key elements*** | | | |
| Key elements included in title  Construct  Population  Type of OMI  Measurement properties  Systematic review | 80  82  66  34  80 |  |  |
| Key elements included in aim  Construct  Population  Type of OMI  Measurement properties | 87  81  76  76 | 94  88  52  81 |  |
| ***Search strategy*** | | | |
| Search strategy matched aim | 78 |  |  |
| Search syntax for at least 1 database provided | 70 |  |  |
| Search appropriate for  Construct  Yes Unclear  No  Population  Yes Unclear  No  Type of OMI  Yes Unclear  No  Measurement properties  Yes Unclear  No | 49  31  20  59  21  20  48  12  40  56  10  34 | 25  50  65  28 |  |
| Number of databases searched, median [range]  MEDLINE  EMBASE | 4 [1-14]  98  56 | 4 [1-15]  92  59 | 93  35 |
| Reference checking used | 66 | 65 |  |
| No time limits used in search or arguments provided for used of time limits | 77 | 72 |  |
| No language restrictions used in search | 66 | 26 | 79 |
| No other notable restrictions used in search | 77 |  |  |
| ***Eligibility criteria*** | | | |
| Inclusion and exclusion criteria clearly defined | 75 | 86 | 72 |
| Eligibility criteria matched aim | 83 |  |  |
| No other notably criteria used in eligibility | 58 |  |  |
| ***Article selection*** | | | |
| Abstract selection by at least 2 independent reviewers  Yes  Partly Unclear  No | 62  3  26  9 | 41  38  21 |  |
| Full-text selection by at least 2 independent reviewers  Yes  Partly Unclear  No | 67  2  27  4 | 38  48  13 |  |
| ***Data extraction*** | | | |
| Data extraction by at least 2 independent reviewers  Yes  Partly Unclear  No | 39  3  44  14 | 25  62  13 | 25  71  4 |
| ***Risk of bias assessment*** | | | |
| Methodological quality assessment of included studies | 63 | 41 | 30 |
| Methodological quality assessment by at least 2 independent reviewers  Yes  Partly Unclear  No | 62  1  33  3 | 60  28  12 |  |
| ***Measurement property evaluation*** | | | |
| Quality of the OMI (measurement properties) evaluated  Yes  Some measurement properties  No | 59  14  27 | 58 | 55 |
| Criteria for measurement properties specified  Yes  For some measurement properties  No | 67  14  19 |  |  |
| Evaluation of each subscale (if multidimensional)  Yes  Partly Unclear  No | 18  5  26  51 |  |  |
| Measurement properties evaluated by at least 2 independent reviewers  Yes  Partly Unclear  No | 21  1  70  8 | 33  62  5 |  |
| ***Data synthesis*** | | | |
| Data synthesis performed (if possible)  Yes  Partly Unclear  No | 57  3  8  31 | 42  58 | 7 |
| Data synthesis performed for each subscale (if multidimensional)  Yes Unclear  No | 13  50  37 |  |  |
| Data synthesis methods clearly described | 47 | 47 |  |
| Data synthesis performed at the level of  Measurement properties  Only domains of measurement properties Only subscales or instruments | 84  13  4 | 79  9  12 |  |
| Data synthesis performed by at least 2 independent reviewers  Yes Unclear  No | 18  75  7 |  |  |
| ***Certainty assessment*** | | | |
| Quality of the evidence graded | 33 |  |  |
| Quality of the evidence graded for each subscale (if multidimensional)  Yes Unclear  No | 15  19  67 |  |  |
| Quality of the evidence graded by at least 2 independent reviewers  Yes Unclear  No | 27  70  3 |  |  |
| ***Presentation of results*** | | | |
| Flow chart provided | 96 |  |  |
| Reasons for excluding full text articles reported  Full information (numbers for each reason) Some information (reasons, but not specifying numbers)  No | 65  20  15 | 55 |  |
| Included instruments in accordance with inclusion criteria  Yes Unclear  No | 86  12  2 |  |  |
| Results of measurement properties reported as raw data  Yes For some measurement properties  No | 42  30  28 | 56  13  31 |  |
| ***Instrument recommendation*** | | | |
| Recommendations for instruments made | 42 | 49 |  |
| Recommendations made for each construct of interest | 25 |  |  |
| OMI recommendation consistent with evidence appraisal  Yes  Partly Unclear  No | 55  7  24  14 |  |  |

** Empty cells indicate a quality aspect was not evaluated in previous reviews*
