# Supplementary material for: Methodological quality of 100 recent systematic reviews of health-related outcome measurement instruments: an overview of reviews
Source: Qual Life Res. 2024 Jul 3;33(10):2593–609. doi: 10.1007/s11136-024-03706-z (PMC11452433; doi:10.1007/s11136-024-03706-z)
Supplement: Supplementary file 3 — Supplementary material 3 (DOCX 17 kb) [file 11136_2024_3706_MOESM3_ESM.docx]

**Supplementary File 3.** Quality appraisal of systematic reviews of outcome measurement instruments and comparisons to previous reviews [9, 10]

| Quality aspect | % this study (n=100) | % 2014 study  (n=102)* | % 2007 study (n=148)* |
| --- | --- | --- | --- |
| ***Key elements*** | | | |
| Key elements included in title  Construct  Population  Type of OMI  Measurement properties  Systematic review | 80  82  66  34  80 |  |  |
| Key elements included in aim  Construct  Population  Type of OMI  Measurement properties | 87  81  76  76 | 94  88  52  81 |  |
| ***Search strategy*** | | | |
| Search strategy matched aim | 78 |  |  |
| Search syntax for at least 1 database provided | 70 |  |  |
| Search appropriate for  Construct  Yes Unclear  No  Population  Yes Unclear  No  Type of OMI  Yes Unclear  No  Measurement properties  Yes Unclear  No | 49  31  20  59  21  20  48  12  40  56  10  34 | 25  50  65  28 |  |
| Number of databases searched, median [range]  MEDLINE  EMBASE | 4 [1-14]  98  56 | 4 [1-15]  92  59 | 93  35 |
| Reference checking used | 66 | 65 |  |
| No time limits used in search or arguments provided for used of time limits | 77 | 72 |  |
| No language restrictions used in search | 66 | 26 | 79 |
| No other notable restrictions used in search | 77 |  |  |
| ***Eligibility criteria*** | | | |
| Inclusion and exclusion criteria clearly defined | 75 | 86 | 72 |
| Eligibility criteria matched aim | 83 |  |  |
| No other notably criteria used in eligibility | 58 |  |  |
| ***Article selection*** | | | |
| Abstract selection by at least 2 independent reviewers  Yes  Partly Unclear  No | 62  3  26  9 | 41  38  21 |  |
| Full-text selection by at least 2 independent reviewers  Yes  Partly Unclear  No | 67  2  27  4 | 38  48  13 |  |
| ***Data extraction*** | | | |
| Data extraction by at least 2 independent reviewers  Yes  Partly Unclear  No | 39  3  44  14 | 25  62  13 | 25  71  4 |
| ***Risk of bias assessment*** | | | |
| Methodological quality assessment of included studies | 63 | 41 | 30 |
| Methodological quality assessment by at least 2 independent reviewers  Yes  Partly Unclear  No | 62  1  33  3 | 60  28  12 |  |
| ***Measurement property evaluation*** | | | |
| Quality of the OMI (measurement properties) evaluated  Yes  Some measurement properties  No | 59  14  27 | 58 | 55 |
| Criteria for measurement properties specified  Yes  For some measurement properties  No | 67  14  19 |  |  |
| Evaluation of each subscale (if multidimensional)  Yes  Partly Unclear  No | 18  5  26  51 |  |  |
| Measurement properties evaluated by at least 2 independent reviewers  Yes  Partly Unclear  No | 21  1  70  8 | 33  62  5 |  |
| ***Data synthesis*** | | | |
| Data synthesis performed (if possible)  Yes  Partly Unclear  No | 57  3  8  31 | 42  58 | 7 |
| Data synthesis performed for each subscale (if multidimensional)  Yes Unclear  No | 13  50  37 |  |  |
| Data synthesis methods clearly described | 47 | 47 |  |
| Data synthesis performed at the level of  Measurement properties  Only domains of measurement properties Only subscales or instruments | 84  13  4 | 79  9  12 |  |
| Data synthesis performed by at least 2 independent reviewers  Yes Unclear  No | 18  75  7 |  |  |
| ***Certainty assessment*** | | | |
| Quality of the evidence graded | 33 |  |  |
| Quality of the evidence graded for each subscale (if multidimensional)  Yes Unclear  No | 15  19  67 |  |  |
| Quality of the evidence graded by at least 2 independent reviewers  Yes Unclear  No | 27  70  3 |  |  |
| ***Presentation of results*** | | | |
| Flow chart provided | 96 |  |  |
| Reasons for excluding full text articles reported  Full information (numbers for each reason) Some information (reasons, but not specifying numbers)  No | 65  20  15 | 55 |  |
| Included instruments in accordance with inclusion criteria  Yes Unclear  No | 86  12  2 |  |  |
| Results of measurement properties reported as raw data  Yes For some measurement properties  No | 42  30  28 | 56  13  31 |  |
| ***Instrument recommendation*** | | | |
| Recommendations for instruments made | 42 | 49 |  |
| Recommendations made for each construct of interest | 25 |  |  |
| OMI recommendation consistent with evidence appraisal  Yes  Partly Unclear  No | 55  7  24  14 |  |  |

** Empty cells indicate a quality aspect was not evaluated in previous reviews*
